# COG6-CDG: Two Novel Variants and Milder Phenotype in a Chinese Patient

**DOI:** 10.1155/2024/9857442

**Published:** 2024-02-12

**Authors:** Xue-Yuan Zhang, Jing Zhang, Yi Lu

**Affiliations:** ^1^The Center for Pediatric Liver Diseases, Children's Hospital of Fudan University, Department of Pediatrics, Shanghai 201102, China; ^2^Shanghai Medical College of Fudan University, Shanghai 201102, China

## Abstract

Here, we present a Han Chinese pediatric girl highly suspected of congenial disorder of glycosylation type IIL (CDG2L; OMIM#614576). Her clinical symptoms include transferase abnormal, liver cirrhosis, hemogram, coagulopathy, growth retardation, intellectual disability, frequent infections, and enamel hypoplasia. Trio-genome sequencing identified in *COG6* a paternal variant c.1672C>T (p.Gln558Ter) and a maternal variant c.153+392A>G (p.?). Reverse transcription-polymerase chain reaction (RT-PCR) using mRNA isolated from peripheral blood confirmed the pathogenicity of both variants. The paternal variant resulted in nonsense-mediated mRNA decay. The maternal variant generated two aberrant *COG6* transcripts with 154 bp overlap and was predicted to result in a frameshift at the same position, leading to generation of a premature termination codon. They might result in synthesis of a truncated form of COG6. Thus, the patient was genetically diagnosed.

## 1. Introduction

Congenial disorder of glycosylation type IIL (CDG2L; OMIM#614576) is an autosomal recessive disorder with a broad clinical spectrum. The core features include liver involvement, developmental disability, recurrent infections, early lethality, hypohidrosis predisposing for hyperthermia, and hyperkeratosis as ectodermal signs [1]. The genetic etiology of CDG2L is variation in *COG6*, encoding the conserved oligomeric Golgi (COG) complex subunit 6. So far, 20 deleterious variants in COG6 were reported [2, 3], including 17 variants located in exons and 3 variants located less than 25 base pairs (bp) away from exon-intron junctions. As known, deep intronic variants (more than 100 bp away from exon-intron junctions) are not uncommon in human diseases [4]. Thus, it is also worth studying such variants as a cause of CDG2L. However, no pathogenic deep intronic variants in *COG6* were reported.

Here, we identified, in a pediatric girl highly suspected of CDG2L, two novel compound heterozygous variants in *COG6* with one nonsense and the other deep intronic variant. Pathogenicity of these two variants was also investigated. This suggested that deep intronic variant in *COG6* should also be considered in *COG6*-CDG.

## 2. Methods

### 2.1. Genome Sequencing (WGS)

Genome sequencing was performed as described [5]. Briefly, genomic DNA (gDNA) was isolated (QIAamp DNA Blood Mini Kit, 51106, Qiagen, Hilden, Germany) from peripheral blood of this patient and her parents. WGS and annotation of gDNA of this trio were done by Aegicare (Shenzhen, China). Genome libraries were constructed using the TruSeq Nano DNA HT Sample prep Kit (Illumina, San Diego, CA) and sequenced on HiSeq 2000 platform (Illumina, San Diego, CA) to generate 150 bp paired end reads at a target depth of 30x. Reads were mapped to reference sequence (GRCh37/hg19) using BWA [6]. SNVs and InDels were called using the HaplotypeCaller module of the Genome Analysis Toolkit (https://software.broadinstitute.org/gatk/best-practices/) [7]. SVs were called using SpeedSeq [8]. Variants were further annotated using ANNOVAR (http://annovar.openbioinformatics.org/en/latest/) [9].

### 2.2. Genetic Analysis

SNVs/InDels and SVs in all glycosylation-related genes were analyzed. To exclude other genetic etiologies, SNVs/InDels in other glycosylation-related genes (including at least *ALG3*, *ALG6*, *COG1*, *COG2*, *COG4*, *OCG5*, *COG6*, *COG7*, *COG8*, *PMM2*, and *SLC35A2*) [10] were also investigated. SNVs/InDels considered pathogenic should meet these criteria as described [5]: (1) allele frequency < 0.01 in the Genome Aggregation Database (gnomAD; https://gnomad.broadinstitute.org/) and in our institutional internal database (data not shown) and (2) located in an exonic/splicing region and predicted to lead to a premature stop codon or to a frameshift insertion or deletion; located in a canonical splice site; or predicted to be pathogenic by at least one of 5 in silico tools including Mutation Taster (http://www.mutationtaster.org/), Polymorphism Phenotyping v2 (http://genetics.bwh.harvard.edu/pph2/), Sorting Intolerant From Tolerant (http://sift.jcvi.org/), Berkeley Drosophila Genome Project (http://www.bdgp.org/), and Human Splicing Finder (HSF; http://www.umd.be/HSF/)). SVs involving coding region with allele frequency < 0.01 in the internal database were considered pathogenic.

### 2.3. Sanger Sequencing and Agarose Gel Electrophoresis

Sanger sequencing in this trio verified the candidate SNVs and their segregation. Primers were designed following standard protocol to amplify sequences flanking the SNVs as well as reference sequences. Targeted polymerase chain reaction (PCR) was conducted according to manufacturer protocols (2× Master Mix KT201; Tiangen, Shanghai, China). GRCh37/hg19 and NM_020751.3 were reference sequences for *COG6*. Sequences for primers were available on demand.

### 2.4. RNA Extraction and Transcript Analysis

PAXgene tubes (PreAnalytiX QIAGEN/BD, Hombrechtikon, Switzerland) were used to collect 2 mL of peripheral blood from this patient and her parents, also a clinically well volunteer. Total RNA was extracted (HiPure PX Blood RNA Kit, Magen, Guangzhou, China) according to the manufacturer's instructions. PrimeScript RT Reagent Kit with gDNA Eraser (RR047A; TaKaRa Bio, Beijing, China) was used for reverse transcription of 0.5 *μ*g RNA into cDNA. RT-PCR analysis was performed to analyze transcripts differentially as described [11]. *COG6*-specific primers were designed to cover the region mutated (Table 1). *COG6* transcripts in the clinically well obligate-heterozygote parents served as controls.

PCR products (35 cycles) were separated by 1% agarose gel electrophoresis. Exons 15-18 of *COG6*, the effected regions of paternal variant, were amplified and subjected to Sanger sequencing (MAP Biotech, Shanghai, China). PCR products of exons 1-3, the effected regions of maternal variant, were analyzed by TA cloning [12] and Sanger sequencing.

### 2.5. Quantitative PCR Analysis

In order to investigate the discrepancy of *COG6* expression in this pedigree, qPCR analysis was performed using the indicated primers (Table 2) on a Roche Light Cycler® II 480 real-time PCR system (Roche, Basel, Switzerland). Primers were designed at regions exon 2-3, exon 14, and exon 19 (the last exon of *COG6*). Primers were verified by in silico PCR (http://genome.ucsc.edu/cgi-bin/hgPcr). The amplicons were obtained using 2×NovoStart® SYBR qPCR SuperMix Plus (E096-01B; Novoprotein, Shanghai, China). PCR conditions were as follows: a preincubation at 95°C for 1 minute (min), 40 cycles of 95°C for 20 seconds (s), 60°C for 20 s, and 72°C for 30 s, followed by a dissociation curve analysis step at 95°C for 5 s, 65°C for 1 min, and 97°C for 15 s to verify the amplification of single and specific products, and a final cooling step to 40°C for 30 s. The relative expression levels for the tested region were determined using glyceraldehyde 3-phosphate dehydrogenase as an internal reference using the comparative Ct (2 × 2^-*ΔΔ*CT^) method. All experiments were conducted in triplicate independently.

## 3. Results

### 3.1. Clinical Phenotype

This girl is the first child of unrelated parents of Han Chinese. No ultrasound anormaly (such as intrauterine growth retardation or microcephaly) was documented in the prenatal period. She was born at term, after a normal pregnancy. She performed well until that fever at 1y5mo old, with peak at 40°C. Routine blood test, at first assessment, showed abnormal aspartate aminotransferase (AST) of 297 IU/L (ref: 0-40), alanine aminotransferase of 74.7 IU/L (ref: 0-40), and platelet value of 67 × 10^9^/L (ref: 150~407). No jaundice was observed. She was observed prone to nausea, vomiting, and choric diarrhea. Infection with syphilis, Epstein-Barr virus, hepatitis virus B and C, parvovirus B19, and human immunodeficiency virus was excluded by serological screening. Ceruloplasmin was 0.18 g/L at first assessment (ref: 0.2-0.6), and no K-F ring was observed. Bone marrow puncture showed deficient megakaryocyte maturation and thrombocytopenia. Her grandfather and an aunt were documented thrombocytopenia. Her aunt also suffers from systemic lupus erythematosus. Liver cirrhosis was suggested by abdominal ultrasound, upper abdominal magnetic resonance imaging, histopathologic studies, and electron microscopy in Women and Children's Medical Center (Guangdong, China).

She was referred to our center, at 2y10mo, for cirrhosis and growth retardation (Supplement Table S1 and Supplement Figure S1). Elevated AST (81.06 U/L) and bile acid (69.9 *μ*mol/L; ref: 0-10). Result of direct antiglobulin test was negative. Anti-liver-kidney microsomal antibody, anti-mitochondria antibody, and anti-smooth muscle antibody were negative, while antinuclear antibody was positive. A score of DQ = 63 (ref: 70) was obtained in Denver Developmental Screening Test. A continued thrombocytopenia was obtained. A liver biopsy revealed noninflammatory cirrhosis. Brain MRI and X-ray test were normal. Clinically, she was highly suspected of congenial disorder of glycosylation.

She is now 3y8mo, with a height of 92 cm and weight of 11.5 kg (Supplement Table S1 and Supplement Figure S1). According to WHO child growth standards (https://www.who.int/tools/child-growth-standards), she has growth retardation. Her head circumference at 3 y is 43 cm, and documented ≤3 SD at 3y8mo by her local hospital. However, when comprehensively considering her height, weight, and circumference, one cannot say the circumference is an anomaly.

### 3.2. Genetic Findings on WGS and Sanger Sequencing

**Figure 1:**
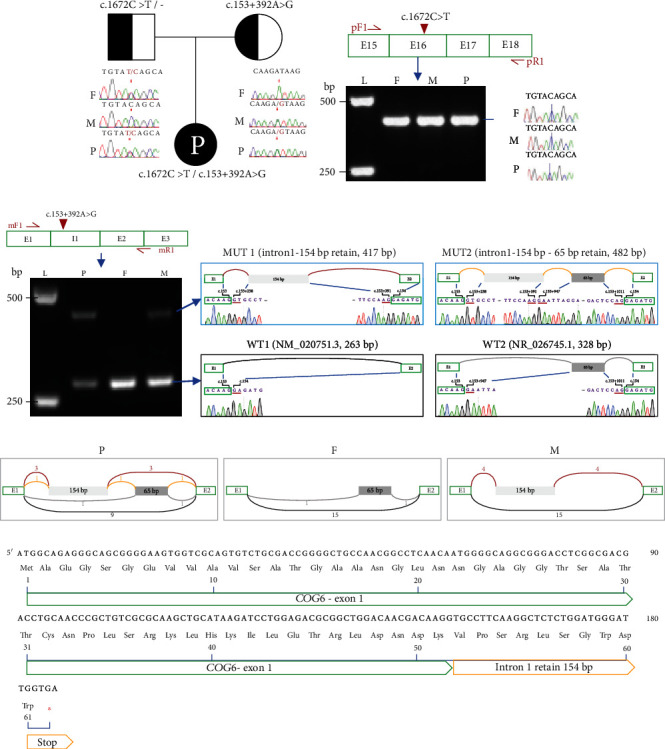
Biallelic variants identified in a Chinese patient and their effects on RNA stability/splicing: (a) pedigree of this family; (b) mRNA region involving paternal variant c.1672C>T (exons 15-18, NM_020751.3) was amplified using primers pF1 and pR1. E: exon; L: DNA ladder; F: father; M: mother; P: patient; (c) mRNA region involving maternal variant c.153+392A>G (exons 1-intron1-exon 2-exon3) was amplified using primers mF1 and mR1. PCR products underwent 1% agarose gel electrophoresis. Splicing pattern and sequences at splicing boundary are shown on right. I: intron; (d) statistics of mRNA splicing pattern using data from 20 TA clones. Numbers indicated clones matched with indicated splicing: NM_020751.3, blue line; NR_026745.1, grey line; MUT1, red line; MUT2, orange line; (e) predicted results of two aberrant splicing products MUT1 and MUT2. Both result in frameshift and a premature stop codon, thus predicted to produce a truncated protein with 61 amino acids.

In this patient, trio-WGS identified in *COG6* biallelic variants: one paternal inherited variant c.1672C>T (p.Gln558Ter) and one maternal inherited variant c.153+392A>G (p.?). Both variants are novel. Sanger sequencing confirmed the two variants and their origin (Figure 1(a)). Pathogenicity of the paternal variant c.1672C>T was designated as pathogenic (PVS1+PM2_supporting) according to American College of Medical Genetics and Genomics (ACMG) guidelines [13], while that of the maternal variant c.153+392A>G is variant of uncertain significance. No pathogenic SNV/SV variants were found in other COG-related genes on WGS.

### 3.3. Paternal Variant c.1672C>T Resulted in Largely Decreased of Mutated RNA

Patient liver suitable for RNA extraction was not available for this patient; however, *COG6* is expressed in blood (https://www.proteinatlas.org/ENSG00000133103-COG6/single+cell+type). Analysis of PBMC mRNA accordingly was undertaken. Variant c.1672C>T (p.Gln558Ter), located on exon 16, was predicted to result in nonsense-mediated mRNA decay. RT-PCR of *COG6* exons 15-18 in this patient and her father produced a single electrophoretogram band identical to wild type (Figure 1(b)). Sanger sequencing of the band confirmed the presence of wild-type *COG6* transcript (ascribed to another allele) in both reverse and forward directions in her father and a noticeable reduction of wild-type COG6 transcript in this patient (Figure 1(b)).

### 3.4. Maternal Variant c.153+392A>G Resulted in Aberrant RNA Splicing

Variant c.153+392A>G, located on intron 1, was predicted as likely to disturb RNA splicing by Splice AI (https://spliceailookup.broadinstitute.org) [14] and varSEAK SSP (https://varseak.bio/) [15]. RT-PCR of *COG6* exons 1-3 in this patient and her mother produced seemingly one additional electrophoretogram band (~500 bp) compared to her father (served as wild-type control, ~263 bp) (Figures 1(c) and 1(d)). Sanger sequencing of the shorter band showed two wild-type *COG6* transcripts: one is coding reference NM_020751.3, spliced as E1-E2 and the other is noncoding reference NR_026745.1, spliced as E1-(c.153+947_c.153+1011)-E2. Sanger sequencing of the longer band also showed two aberrant transcripts: one harboured 154 bp of intron 1, spliced as E1-(c.153+237_c.153+391)-E2, and was designated as MUT 1 and the other involves the 154 bp together with an additional 65 bp of intron 1, spliced as E1-(c.153+237_c.153+391-c.153+947_c.153+1011)-E2, and was designated as MUT 2. Both aberrant transcripts resulted in frameshift and a premature termination codon at the same site (31 bp after E1), thus predicted to result in a truncated protein with 61 amino acids (Figure 1(e)).

Statistical analysis of 20 clones showed that the aberrant transcripts were only detectable in the girl and her mother (Figure 1(d)) and account for 21%~25% (4/16 in the girl and 4/19 in her mother). Further, MUT1 may be the mainly aberrant isoform (MUT1 : MUT2 = 3 : 1 in the girl and 4 : 0 in her mother).

### 3.5. COG6 Expression Is Comparatively Similar in This Girl than Her Parents

**Figure 2:**
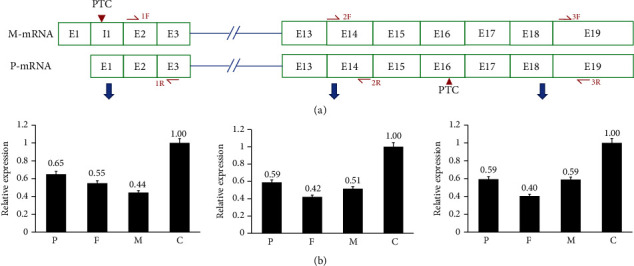
Relative *COG6* expression in this family: (a) illustration of two kinds of mRNAs in this patient, resulted from maternal variant and paternal variant. 1F/1R, 2F/2R, and 3F/3R are three pairs of primers used in quantitative PCR (qPCR). M-mRNA: maternal mRNA; P-mRNA: paternal mRNA; PTC: premature termination codon; (b) qPCR results show that quantity of *COG6* mRNA in this patient is not the lowest in her family (three independent experiments for each primer pair). P: patient; F: father; M: mother; C: control.

*COG6* expression in this girl is not greatly decreased than her parents (Figure 2), while at a relatively same level (0.5 ± 0.2). Same results were obtained using primers at the head, the waist, and the tail region of mRNA. Considering the results of TA clones (Figures 1(c) and 1(d)), differences existed in the composition of transcripts in this girl. A higher percentage of aberrant RNAs may be expressed in the girl.

## 4. Discussion

*COG6*-CDG disease is a rapidly growing family with 43 cases reported [3]. They show a great phenotypic variability ranging from homozygous intronic variants c.1167-24A>G as the very mild form to loss-of-function variants leading to early lethal [1]. Among the 32 cases individually clinically described, the core features of *COG6*-CDG including liver involvement (23/32), hypotonia (22/32), microcephaly (22/32), developmental delay/intellectual disability (21/32), abnormal brain MRI (20/32), hypohidrosis (18/32), thrombocytopenia/pancytopenia/coagulopathy (14/32), gastrointestinal disorder (10/32), recurrent infections (8/32), and early lethality (4 d~15 mo, 17/32). This girl in this study had an early onset at 6 mo and presented features of growth retardation (height < 3%, weight < 3%, and DQ 63); abnormal liver function with nodular cirrhosis, bile duct injury, and mild inflammation (G1-2S4); thrombocytopenia/coagulopathy; and prone to nausea/diarrhea. Hypotonia and microcephaly are prominent *COG6*-CDG features. Monitoring of this girl for skin and neurological disease may be in order.

Variants in deep intronic are either missed by exome sequencing or were classified as unknown significance by regular variants interpretation pipeline at first met. Variants at >100 and even >1000 bp far away from exons are proved to be pathogenic through combining of genome sequencing and RNA study [16–19]. Such variants usually exert a pathogenic effect by causing pseudoexon inclusion into the transcript, further being translated into damaged protein or experience NMD.

There are, including the girl presented in this study, 22 pathogenic variants reported. Among them, 5 were noncanonical splicing: c.540G>A, c.518_540+3del, c.1075-9T>G, c.1167-24A>G, and c.153+392A>G. This accounts for 22.7%, suggesting that pathogenicity should be considered on such variant in patients with one pathogenic variant. Previously, pathogenicity was only experimentally evaluated for c.1167-24A>G [20]. RT-PCR analysis, using skin fibroblast-derived RNA from, revealed a 75% aberrant transcript with 37 bp intronic retention of intron 12, resulting in a frameshift and insertion of a premature stop codon (p.Gly390Phefs∗6).

In this study, the effect of c.153+392A>G was investigated using blood-derived RNA. RT-PCR and TA cloning analysis revealed two aberrant transcripts due to one or two splicing sites in intron 1. With an overlap of 154 bp pseudoexon, both transcripts was predicted to lead to an insertion of 10 amino acids followed by a premature stop codon (p.Glu52Valfs∗10), or experience nonsense-mediated mRNA decay (NMD). This proof makes it be classified as pathogenic (PVS1+PM2_Supporting+PM3+PP4). Combined with a paternal pathogenic variant c.1672C>T (p.Gln558Ter), this girl was genetically diagnosed. Experiences on this case warranted a worth of RNA analysis on a variant in deep intron of *COG6*.

Of note, the total mRNA level of this girl is not lower than her healthy parents (qPCR result 0.5 ± 0.2 is acceptable); reasons may exist that (1) *COG6* expression is more active in toddler than in adults; (2) compensatory producing of *COG6* in this girl, but with unbalanced NMD, thus accumulated; and (3) variant c.153+392A>G is partially penetrant, a mechanism reported accounting for pathogenicity caused by intronic variants [19]. It was also reported in COG6 patients with biallelic homozygous c.1167-24A>G [20], with ~25% normal transcript detected. This, to some extent, could explain the relatively mild phenotype of this girl. Research in homozygous c.153+392A>G patients, if available, may help for exact calculation of penetrance. Limitation of this study is without information for transferrin pattern and serum N- and O-glycosylation structural analyses, due to instruments and technicians not available.

In conclusion, this study reported two novel variants in *COG6*, one nonsense and one deep intronic variant, and proved their pathogenicity, expanding the genetic spectrum of CDG2L. Whole genome sequencing and RNA study permitted precise diagnosis and genetic counselling. When deep intronic variants are found in CDG-related genes, strongly implicated by clinical assessment, one should consider using RNA study tools in the analysis of its pathogenicity.

## Figures and Tables

**Table 1:** Primers used for effects of variants on RNA decay or splicing.

Amplified region	Primer name	Sequence (5′-3′)	Length of PCR product (WT^a^/Mut)	
*COG6_exons 15-18*	c1672_F	TCATGTGTCTTGGATCCTCTCC	402 bp	402 bp
	c1672_R	CACTGTGGCACTTAGAAGAAAGT		

*COG6_exon 1-3*	c153_F	TGCCAACGGCCTCAACAAGG	263 bp	417 bp
	c153_R	TGCTTTCAAGTTCCTCCTTCACT	328 bp	482 bp

^a^There are two wild-type (WT) manuscripts (NM_020751.3, 263 bp, and NR_026745.1, 328 bp), owing to an additional splicing site in intron 1.

**Table 2:** Primers used for qPCR.

Target gene region	Primer name	Sequence (5′-3′)
*COG6 (exon 2-3)*	COG6-E2_F	GCTCTCAAGGCACTTTCAACC
	COG6-E2_R	TGCTTTCAAGTTCCTCCTTCACT

*COG6 (exon 14)*	COG6-E14-F	GTTGAACTCCCACCACCTGAT
	COG6-E14-R	TGCACAAAATCAGCTTGACGA

*COG6 (exon 19)*	COG6-E19-F	TGAAGTGTATGCAGCCGTGA
	COG6-E19-R	ACCTTCAGTGTTTTAGGGAGGT

*GAPDH*	GAPDH-F	GCGAGATCCCTCCAAAATCAA
	GAPDH-R	GTTCACACCCATGACGAACAT

## Data Availability

All data relevant to the study are included in the article. We have submitted our variation to ClinVar with accession numbers SCV003915431 for c.1672C>T and SCV003915432 for c.153+392A>G. The girl's height and weight in details at different ages are shown in Supplementary files (Figures S1 and Table S1).

## Supplementary Materials

Supplementary 1 Supplement Table S1: height, weight, and circumference of this girl at different ages.

Supplementary 2 Figure S1: height and weight of this girl at different ages. (A) shows that this girl has growth retardation with *Z*-scores at about -2, using the girl's chart—length/height-for-age; (B) shows that this girl has low weight with −3 < *Z* − scores < −2, using the girl's chart—weight-for-age (according to the WHO child growth standards (https://www.who.int/tools/child-growth-standards). *Z*-scores are used to describe how far a measurement is from the median (average). Black dots show the position of the girl.
