# Supplementary material for: COG6-CDG: Two Novel Variants and Milder Phenotype in a Chinese Patient
Source: Hum Mutat. 2024 Feb 12;2024:9857442. doi: 10.1155/2024/9857442 (PMC11919040; doi:10.1155/2024/9857442)
Supplement: Supplementary 1 — Supplement Table S1: height, weight, and circumference of this girl at different ages. [file 9857442.f1.docx]

| Supplement Table S1. Height, weight and circumference of this girl at different ages | | | |
| --- | --- | --- | --- |
| Age | Height  (cm) | Weight  (kg) | Circumference  (cm) |
| on birth (37w5d) | - | 2.7 | - |
| 1y5mo | 73 | 8 | - |
| 2y | 80 | 8.5 | - |
| 2y10mo | 82 | 9.6 | - |
| 3y | 87.9 | 10 | 43 |
| 3y8mo | 92 | 11.5 | - |
| cm, centimeter; kg, kilogram; w, weeks; d, days; y, years; mo, months  -, not measured. | | | |
