# Supplementary material for: COG6-CDG: Two Novel Variants and Milder Phenotype in a Chinese Patient
Source: Hum Mutat. 2024 Feb 12;2024:9857442. doi: 10.1155/2024/9857442 (PMC11919040; doi:10.1155/2024/9857442)
Supplement: Supplementary 2 — Figure S1: height and weight of this girl at different ages. (A) shows that this girl has growth retardation with Z-scores at about -2, using the girl's chart—length/height-for-age; (B) shows that this girl has low weight with −3 < Z − scores < −2, using the girl's chart—weight-for-age (according to the WHO child growth standards (https://www.who.int/tools/child-growth-standards). Z-scores are used to describe how far a measurement is from the median (average). Black dots show the position of the girl. [file 9857442.f2.docx]

| A. |
| --- |
| 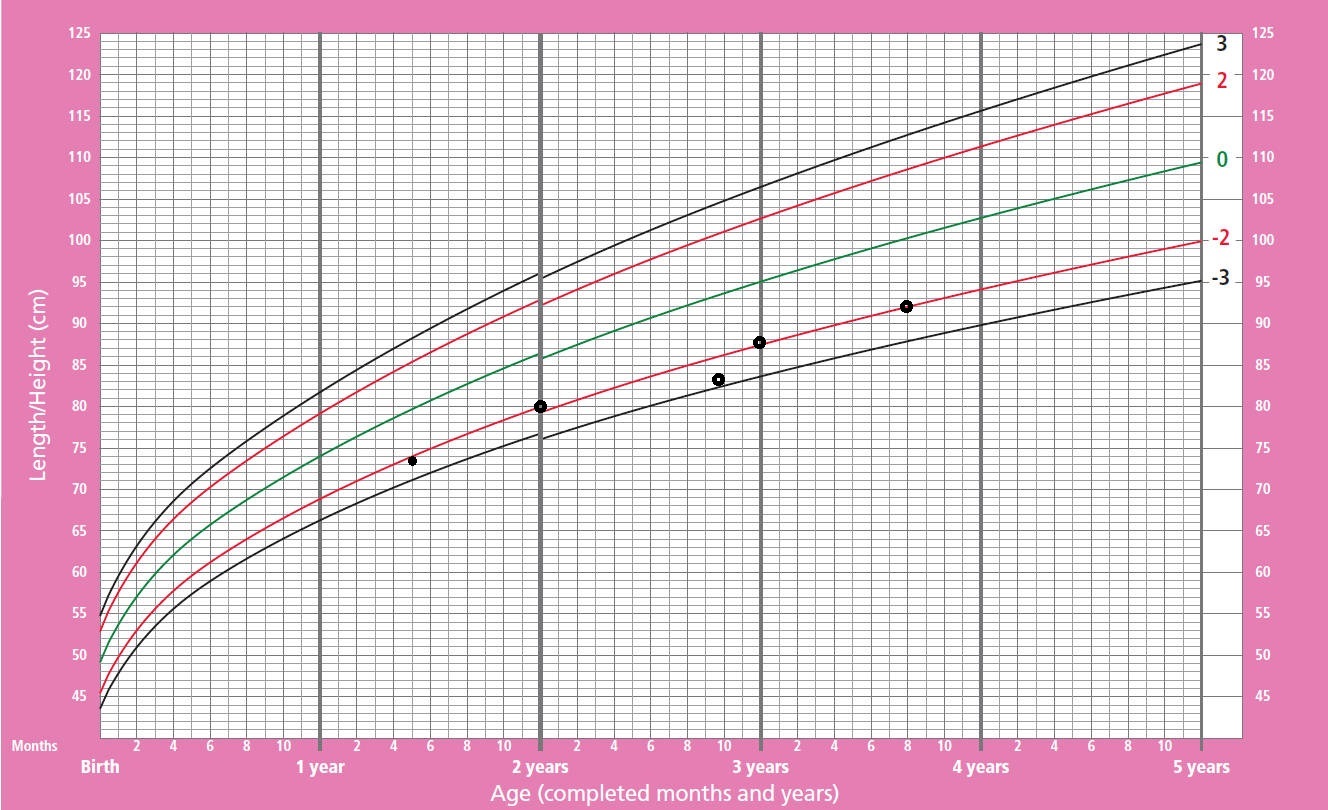 |
| B. |
| 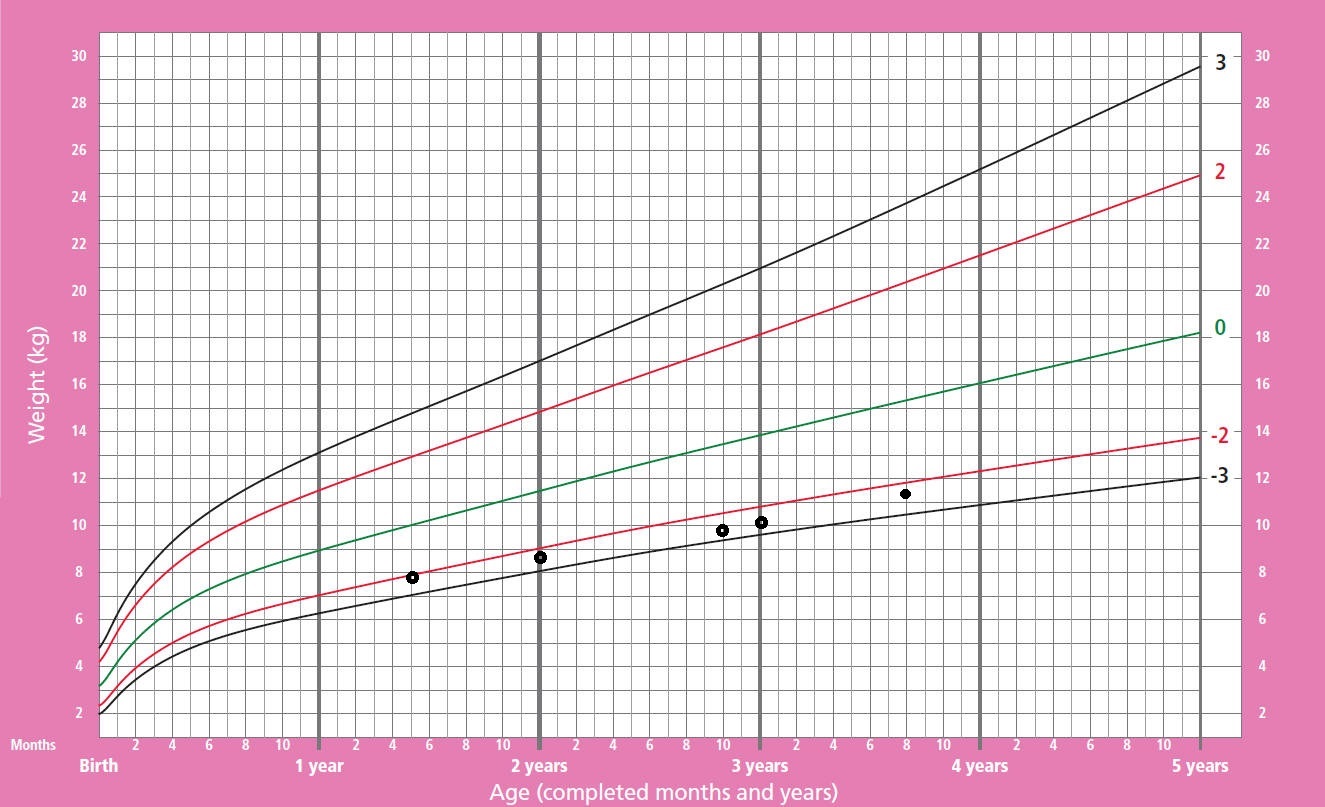 |
| Figure S1. Height and weight of this girl at different ages.  (A) shows this girl has growth retardation with Z-scores at about -2, using girls chart-length/height-for-age; (B) shows this girl has low weight with -3 < Z-scores < -2, using girls chart-weight-for-age. According to the WHO child growth standards ([**https://www.who.int/tools/child-growth-standards**](https://www.who.int/tools/child-growth-standards) ). Z-scores are used to describe how far a measurement is from the median (average). Black dots shows the position of the girl. |
